# The Significance of Relative Cerebral Blood Volume Index in Discriminating Glial Tumors from Brain Metastasis Using Perfusion Magnetic Resonance Imaging

**DOI:** 10.3390/diagnostics15111324

**Published:** 2025-05-25

**Authors:** Ayşe Eda Parlak, Burak Yangoz

**Affiliations:** Department of Radiology, Health Sciences University, Antalya Training and Research Hospital, Antalya 07100, Turkey; yangozburak@gmail.com

**Keywords:** perfusion, MRI, glial tumor, metastasis, rCBV, brain

## Abstract

**Background/Objectives:** The accurate diagnosis and classification of brain tumors are critical for appropriate treatment planning and patient management. We evaluated the effectiveness of perfusion in differentiating glial tumors from metastases using dynamic susceptibility-weighted contrast enhanced perfusion MRI (DSC-MRI) **Methods:** A total of 95 consecutive patients with pathological diagnoses of brain tumors who underwent perfusion MRI between July 2021 and March 2023 were retrospectively recruited. Conventional and perfusion MRI were evaluated, and tumoral and peritumoral relative cerebral blood volume (rCBV) values were measured. Mann–Whitney U and Kruskal–Wallis tests were performed for non-parametric comparisons of continuous data. The optimal cut-off value of rCBV in differentiating tumor types was evaluated with the receiver operating characteristic (ROC) curve analysis. **Results:** Tumoral rCBV (*p* < 0.001) and peritumoral rCBV values (*p* = 0.001) were significantly higher in glial tumors than metastases. Further subgroup analyses showed that tumoral and peritumoral rCBV values of glial tumors were higher than those of non-small-cell lung cancers (*p* < 0.001 and *p* = 0.003, respectively) and those of breast cancer (*p* = 0.311 and *p* = 0.053, respectively) in discriminating high-grade glial tumors and metastases. ROC analyses showed that area under the curve values for tumoral and peritumoral rCBV were 0.816 and 0.725, respectively, for the optimal cut-off points 1.339 and 1.238 (87.5% and 58.33% sensitivity; 73.85% and 90.77% specificity, respectively). Multivariate analysis showed that increased tumoral rCBV and peritumoral rCBV values were independent risk factors for glial tumor occurrence. **Conclusions:** DSC-MRI is an effective method to differentiate glial tumors and metastases. Higher rCBV values may serve as a determinant for the diagnosis of glial tumors and metastatic brain tumors.

## 1. Introduction

The accurate diagnosis and classification of brain tumors are critical for appropriate treatment planning and patient management. Over the years, there has been a growing interest in the use of advanced imaging techniques, including perfusion magnetic resonance imaging (MRI) as an auxiliary tool in the differentiation of brain masses, which are commonly encountered tumors in adults [1,2,3,4]. The assessment of relative cerebral blood volume (rCBV) values in tumors is performed by perfusion MRI, providing valuable information about tumor vascularity and angiogenesis [3,5,6,7].

Numerous studies have been conducted to investigate the diagnostic performance of perfusion MRI in discriminating glioma from brain metastasis [5,8,9]. These studies utilized various MRI protocols and parameters to evaluate the rCBV as a potential biomarker for distinguishing these types of brain tumors [4,10,11]. Past studies investigated the significance of a higher rCBV value, as a measure of the perfusion MRI, in evaluating diagnostic accuracy in tumor recurrence in addition to initial diagnosis [4,12]. Perfusion MRI relies on the principle that hypervascular tumors, particularly high-grade gliomas, exhibit increased angiogenesis and blood vessel density compared to normal brain tissue [3,6]. Also, peritumoral rCBV is evaluated due to the fact that the presence of neoplastic cells infiltrating the peritumoral region has been documented within the peritumoral edema surrounding primary high-grade gliomas [7].

Gliomas, originating from the central nervous system (CNS), and brain metastases, secondary to systemic malignancies, pose distinct diagnostic challenges [13]. Since both tumor types often present similar radiological patterns, it is challenging to distinguish them solely based on conventional MRI sequences [14,15,16,17]. However, their underlying pathophysiology diverges significantly, particularly in terms of vascularity and tumor microenvironment [18]. In similar findings in conventional MRI, high-grade gliomas exhibit intricate angiogenetic properties and manifest as elevated perfusion characteristics, whereas brain metastases typically display lower perfusion rates and discrete margins [14]. However, some metastatic tumors, due to their high vascularity and consequently elevated perfusion values, can mimic high-grade gliomas [1,4,9]. The rCBV values derived from perfusion MRI demonstrated the potential to provide valuable insights in vascularization patterns, extension, and the nature of intracranial tumors [5].

To the best of our knowledge, no past studies subgrouped metastasis types and compared primary glial tumors.

This study aims to investigate the role of dynamic susceptibility-weighted contrast enhanced perfusion MRI (DSC-MRI) in differentiating primary glial tumors and metastatic brain tumors based on the primary site of origin using tumoral and peritumoral rCBV values.

## 2. Materials and Methods

This study was approved by the IRB Committee of the University of Health Sciences Antalya Training and Research Hospital (2023/076) and was conducted in accordance with the 1964 Declaration of Helsinki. Patient records and information were anonymized and de-identified prior to analysis. The requirement for informed consent was waived by the ethics committee of the University of Health Sciences Antalya Training and Research Hospital due to the retrospective nature of the study.

### 2.1. Patient Selection

In this retrospective study, we enrolled 95 consecutive patients with histopathological diagnoses of glial tumors and metastatic brain tumors who underwent MRI perfusion examinations at our medical facility then underwent surgical procedures between July 2021 and March 2023. Patients from the neurosurgery and neurology clinics were selected by the physicians and referred to the radiology department for a brain MRI perfusion study in patients with brain masses prior to surgery. The histology results were obtained through the patient’s clinical database. The WHO classification of the primary CNS tumors was determined by the pathologist based on the “2021 WHO Classification of Tumors of the Central Nervous System” [19].

MR images of patients were retrieved from the picture archive and communication system (PACS) database. Additionally, demographic characteristics (age and gender) were recorded. Exclusion criteria were age < 18, history of major brain trauma, prior surgery, and technical inadequacy, including the presence of metallic artifacts, uncontrolled breathing, or patient movement resulting in image degradation.

### 2.2. Magnetic Resonance Imaging

MR images were acquired by using a multichannel superconducting 3T MRI scanner (Philips Ingenia, Philips Medical System Nederland B.V., Eindhoven, The Netherlands) equipped with high-speed gradients. A dStream HeadNeck Array coil with 16 receive channels was used for the scanning. The MRI protocol was composed of diagnostic focused whole-brain MRI sequences including axial and sagittal turbo spin-echo T2-weighted (TR/TE: 3000/80; scan thickness 5 mm; slice gap 1 mm), axial-fluid-attenuated inversion recovery (FLAIR) (TR/TE: 11,000/120; scan thickness 5 mm; slice gap 1 mm), 3D T1 weighted (TR/TE: 25/1.74; scan thickness 2 mm; slice gap 0.4 mm) and subsequent diffusion-weighted images and susceptibility-weighted imaging (SWI) for all participants. DSC-MRI was used with a first-pass, contrast-enhanced, T1-weighted, single-shot, gradient-echo, echo-planar sequence using a rapid bolus (5 mL/s) of 0.2 mmol/kg of contrast material through an 18 or 20 G intravenous line. Enhanced 3D images were obtained after perfusion-weighted images. The parameters of the sequence were TR/TE; 2000/54; a section thickness of 5 mm, a section gap of 1 mm, a FOV of 224 × 224 × 100 mm, and a flip angle of 30°. All sequences had a number of excitations (nex) of 2.

### 2.3. Image Interpretation

For diffusion weighted imaging, the restricted diffusion term is used for lesions showing a hyperintense signal in diffusion-weighted imaging as well as a hypointense signal on the ADC map [20].

For DSC-MRI, the MR data were then transferred to a separate workstation and processed using commercially available postprocessing software (Phillips, Extended MR workspace, 2.6.3.2.HF3, Best, The Netherlands) based on the previously described algorithm [16].

The arterial input function was manually defined in the middle cerebral artery of the unaffected contralateral hemisphere, and multiparametric perfusion maps were calculated for DSC. rCBV maps were then created from DSC perfusion datasets. All the quantification maps acquired were normalized to the unaffected contralateral brain parenchyma. Then measurements were performed using a total of four ROIs drawn on the enhanced T1-weighted images, also taking into consideration the rCBV maps—one from the peritumoral region, one from the contrast-enhancing tumoral area, and two from the contralateral tumor-free brain parenchyma corresponding to these regions. The peritumoral region was defined as the area within one centimeter outside the external tumor margin [16].

**Figure 1:**
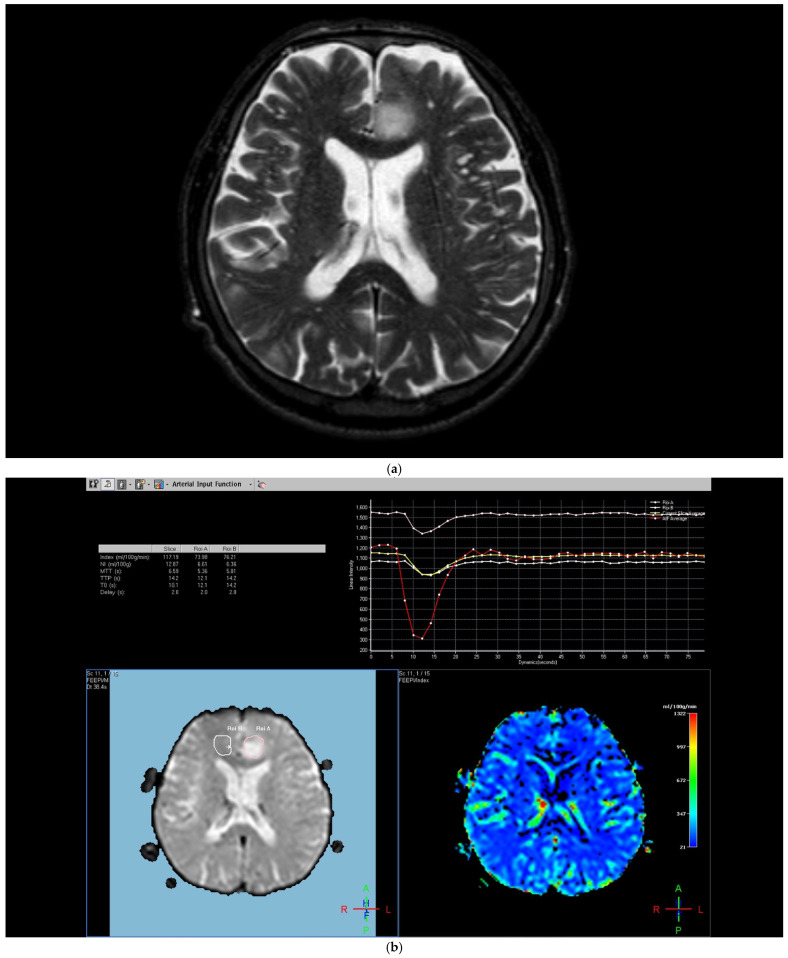

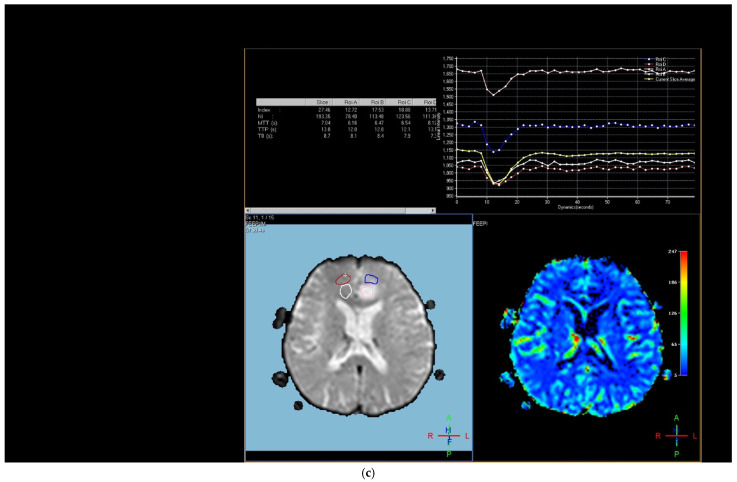
Brain magnetic resonance imaging (MRI) of a 52-year-old male with a low-grade glial tumor revealed (**a**) increased signal on a T2-weighted image, (**b**) hypoperfusion with a rCBV of 1.08 in perfusion-weighted imaging, Pink ROI is from tumor and white ROI is from contralateral parenchyma. and (**c**) a total of four ROIs—one from the peritumoral region (in blue), one from the contrast-enhancing tumoral area (in pink), and two from the contralateral tumor-free white matter areas corresponding to these regions (in white and red) in perfusion-weighted imaging.

The region of interest (ROI) size ranged from 2 to 10 square millimeters in area, depending on the size of the tumor. rCBV values were obtained by identifying regions of maximal perfusion from color maps (Figure 1). While measuring the rCBV values, care was taken to avoid any vascular structures, cerebrospinal fluid, or areas with high susceptibility. For the appropriate placement of the ROI, the rCBV maps were coregistered with FLAIR/T2-weighted, post-contrast T1-weighted, or SWI/GRE images. For multiple metastatic lesions, the measurements were performed from the largest and most enhanced tumoral lesion.

The rCBV values within the tumor and the surrounding area of edema were calculated using the following equation: rCBV = CBV tumoral or peritumoral edema/CBV of the corresponding locations at the uninvolved contralateral brain parenchyma. Perfusion curves were obtained for each lesion. All measurements were performed by the same neuroradiologist (AEP) blinded to the histological diagnosis of the tumors.

### 2.4. Statistical Analyses

Continuous data are represented as median values with percentiles (25–75 percentiles), and categorical variables are presented as frequencies (*n*) and percentages (%). Normality assumption was controlled by the Shapiro–Wilk test. Pearson chi-square and Fisher’s Exact tests were used to compare categorical variables. The Mann–Whitney U test and Kruskal–Wallis test were performed for non-parametric comparisons of continuous data. Post hoc analysis was performed using Bonferroni correction. The optimal cut-off value of rCBV differentiating primary glial tumors and metastases was evaluated with the receiver operating characteristic (ROC) curve analysis. The area under the curve (AUC), sensitivity, and specificity were calculated and reported with 95% confidence intervals. Multivariate logistic regression analysis was used to establish which predictor variables were significantly related to determine factors associated with glial tumors. All patients with complete data for the variables included in the multivariate analysis were retained in the model. Cases with missing values were excluded from the respective analyses. The multivariate logistic regression model was adjusted for the following variables: age, tumor size, heterogeneous contrast enhancement, SWI (microhemorrhage), tumoral rCBV, and peritumoral rCBV. Odds ratios (ORs) with corresponding 95% confidence intervals (95% CIs) are reported. All statistical analyses were carried out using IBM SPSS Statistics for Windows, Version 23.0 (IBM Corp., Armonk, NY, USA). A value of two-sided *p* < 0.05 was considered statistically significant.

## 3. Results

In this study, 95 consecutive patients were enrolled [53 males (55.8%) and 42 females (44.2%); median age of 60 years (interquartile range: 51–67) ranging between 22 and 83]. Within this cohort, 50 (52.6%) of patients were found to have a solitary tumor. The median tumor dimensions measured 20 mm (IQR: 12–34 mm). The predominant tumor location was supratentorial (*n* = 53; 55.8%). Notably, contrast-enhanced imaging patterns were primarily peripheral 44 (46.3%) or heterogeneous 31 (32.6%). Restricted diffusion was observed in 38 (40%) of the patient cohort, and 38 (40%) of cases revealed microhemorrhage in susceptibility-weighted imaging (SWI) (Table 1).

Regarding perfusion metrics, the median rCBV within the tumoral regions was calculated as 1.005 (IQR: 0.663–2.032), while the rCBV within peritumoral areas yielded a median value of 0.915 (IQR: 0.654–1.202). Among the patient cohort, 30 individuals (31.6%) were diagnosed with primary glial brain tumors [6 (6.3%) were low-grade glial tumors, 24 (25.3%) were high-grade glial tumors]. A total of 40 patients (42.1%) had metastatic lung tumors [5 (5.3%) were small-cell and 35 (36.8) were non-small-cell-type)], 12 patients (12.6%) had metastatic breast tumors, and 13 patients (13.7%) had metastatic tumors originating from other organs (Table 1).

The median age of patients with glial tumors was significantly lower than that of patients with brain metastases (*p* = 0.001). Gender (*p* = 0.097) and diffusion properties (*p* = 0.176) were similar between the two groups. Single tumors were significantly higher in patients with glial tumors (93.3% and 33.8%), whereas multiple tumors were significantly higher in patients with brain metastases (26.2% and 0%) (*p* < 0.001). Concomitant supratentorial and infratentorial tumor localization was significantly higher in patients with brain metastases (44.6% and 0%), while supratentorial tumor localization was significantly higher in patients with glial tumors (93.3% and 38.5%) (*p* < 0.001). Heterogeneous contrast enhancement was significantly higher in glial tumors (66.7% and 16.9%), whereas peripheral contrast enhancement was significantly higher in brain metastases (63.1% and 10%) (*p* < 0.001). The presence of microhemorrhage in SWI was significantly higher in the glial tumor group (*p* = 0.024). Median tumoral rCBV (*p* < 0.001), and peritumoral rCBV values (*p* = 0.001) were significantly higher in the glial tumor group than those of metastatic tumors (Table 2).

**Figure 2:**
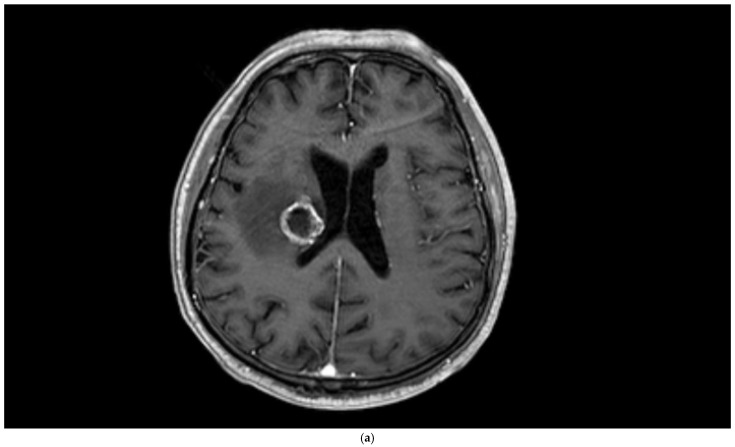

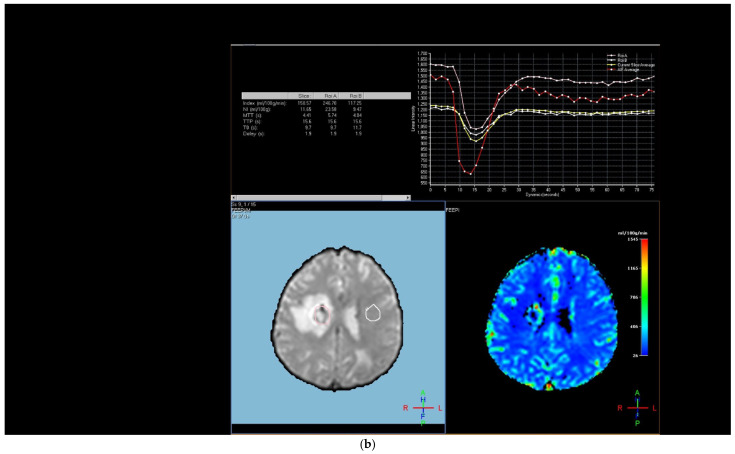
Brain magnetic resonance imaging (MRI) of a 67-year-old male with small-cell lung carcinoma revealed (**a**) peripherally enhanced right periventricular mass on post-contrast axial T1-weighted image and (**b**) hyperperfusion with a rCBV of 2.4 in perfusion-weighted imaging. Pink ROI is from metastatic nodule and white ROI is from contralateral parenchyma.

We further analyzed tumoral and peritumoral rCBV values in detail in terms of the primary tumor origin. There were significant differences in tumoral rCBV (*p* < 0.001) and peritumoral rCBV (*p* = 0.004) values between all primary tumors and all metastatic tumors. When we further broke down the analyses based on the type of the primary tumors, tumoral and peritumoral rCBV values of patients with primary brain tumors were significantly higher than those of patients with lung (*p* < 0.001 and *p* = 0.041, respectively) and metastases from miscellaneous organs (*p* = 0.009 and *p* = 0.004, respectively) (Figure 2) (Table 3).

**Figure 3:**
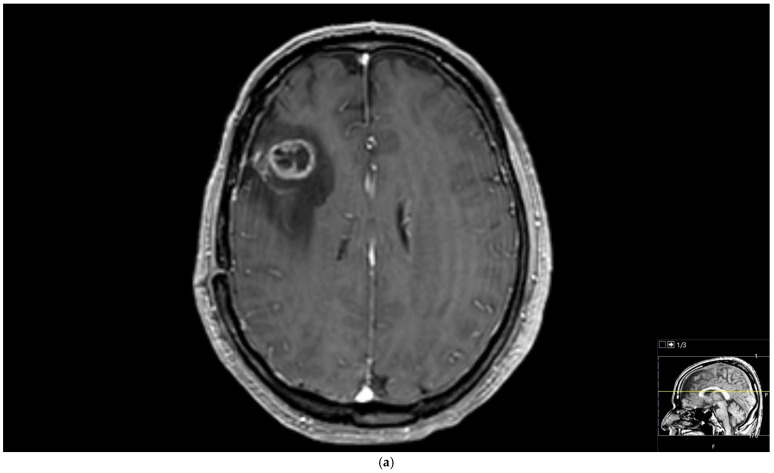

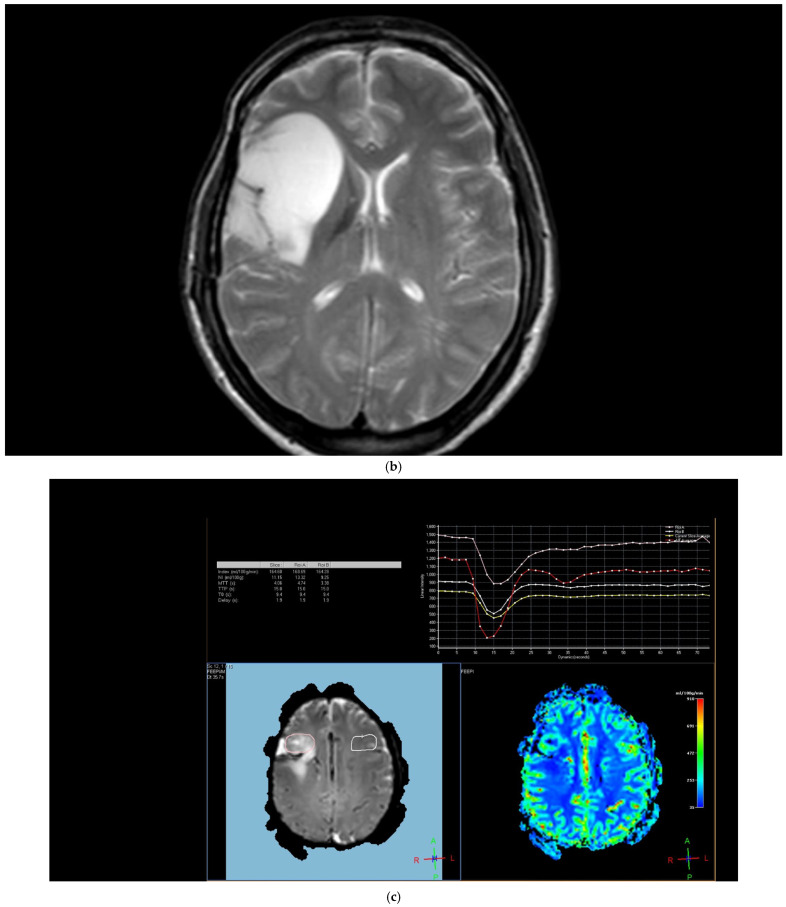
Brain magnetic resonance imaging (MRI) of a 51-year-old male histopathologically diagnosed with glioblastoma. (**a**) Increased signal on T2-weighted image with peritumoral edema, (**b**) peripherally enhanced right frontal subcortical mass on post-contrast axial T1-weighted image, and (**c**) hyperperfusion with a rCBV of 1.5 in perfusion-weighted imaging (Pink ROI is from tumor and white ROI is from contralateral parenchyma).

We further performed analyses based on the grade of the glial tumors. Tumoral rCBV values of high-grade glial tumor patients were significantly higher than those with low-grade glial tumors (Figure 3) (*p* = 0.009). Tumoral and peritumoral rCBV values of patients with high-grade glial tumors were higher than those with brain metastases (Figure 3) (*p* < 0.001 and *p* = 0.001). For the lung cancer types, although statistically not significant, there was a trend toward higher values in tumoral rCBV of high-grade glial tumors than small-cell lung cancer patients (*p* = 0.068). Tumoral and peritumoral rCBV medians of high-grade glial tumor patients were significantly higher than those of non-small-cell lung cancer patients (*p* < 0.001 and *p* = 0.003). While the median rCBV in both the tumoral and peritumoral regions of patients with high-grade glial tumors exceeded that of patients with breast cancer, the difference did not attain statistical significance (*p* = 0.311 and *p* = 0.053). Tumoral and peritumoral rCBV values were higher in patients with high-grade glial tumors compared to metastasis from miscellaneous organs (*p* < 0.001 and *p* = 0.003).

**Figure 4:**
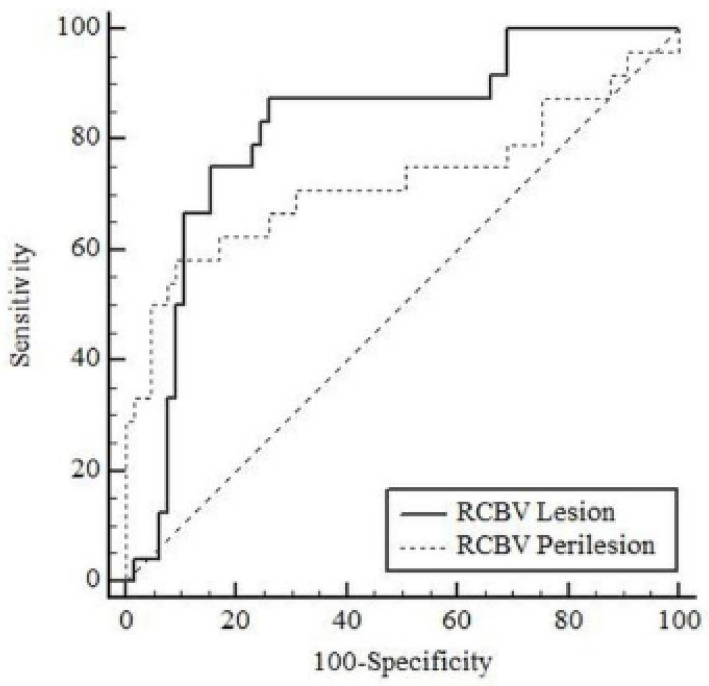
Receiver operating characteristics curve for relative cerebral blood volume values in differentiating high-grade glial tumors from brain metastases (*n* = 89).

Table 4 shows the results of ROC analysis for tumoral rCBV and peritumoral rCBV in differentiating high-grade glial tumors from brain metastases in patients. The AUC value for tumoral rCBV was 0.816 (95% CI: 0.720–0.890; *p* < 0.001) and for peritumoral rCBV was 0.725 (95% CI: 0.620–0.814; *p* = 0.003) in differentiating high-grade glial tumors from brain metastases (Figure 4). According to the Youden index, the optimal cut-off point for tumoral rCBV was >1.339 (sensitivity: 87.5%, specificity: 73.85%) and for peritumoral rCBV was >1.238 (sensitivity: 58.33%, specificity: 90.77%).

Multivariate logistic regression analysis was performed to determine the risk factors independently associated with high-grade glial tumor presence as shown in Table 5. Since tumoral rCBV and peritumoral rCBV values were associated, two separate models were established. The risk of occurrence for high-grade glial tumors increased with the increase in rCBV tumoral (OR: 2.321; 95% CI: 1.155–4.663; *p* = 0.018) and peritumoral rCBV values (OR: 5.07; 95% CI: 1.161–22.137; *p* = 0.031).

## 4. Discussion

The brain hosts hematologic metastases from other organs as well as primary malignancies. Metastatic brain tumors constitute approximately 30–50% of supratentorial brain tumors and stand out as an important health problem [2,4,5]. The differentiation of these two pathologic entities is critical in the clinical management of patients, since treatment and follow-up algorithms differ considerably [2]. Although conventional MRI is one of the most widely used methods in the diagnosis of brain tumors, it is often insufficient to distinguish between these lesions using conventional sequences [2]. Especially in single lesions, the diagnostic accuracy is quite low with conventional methods, and therefore there is a need for advanced diagnostic methods. The literature showed that tumoral rCBV values of metastatic and primary brain tumors obtained from perfusion MRI are significantly different [1,2,8]. Furthermore, past studies revealed that not only peritumoral T2 high signal areas but also normal appearing peritumoral brain tissue had perfusion abnormalities, indicating that there is invisible tumoral infiltration within this area [2]. Also, it has been reported that peritumoral rCBV values of metastases and glial tumors are different from each other [1,2,21]. Vasogenic edema and increased signal on T2-weighted images are observed around metastatic lesions due to increased capillary endothelial permeability and impairment of the blood–brain barrier [22]. Different from the metastases, in high-grade glial tumors, peritumoral T2 signal enhancement occurs secondary to partial peritumoral infiltration as well as disruption of the blood–brain barrier [23]. Hence, perfusion MRI has become a promising tool in the differentiation of these lesions, as it allows the visualization of neovascularization and microvascularization independently of blood–brain barrier disruption. Past studies revealed that the differentiation of glioblastoma and brain metastases shows especially high diagnostic performance with a sensitivity and specificity of over 90% It is widely used in combination with other advanced diagnostic modalities in the differential diagnosis of contrast-enhancing brain lesions worldwide. It has been reported that peritumoral rCBV values increased the sensitivity and specificity of the perfusion MRI [5]. In the current study, both median tumoral and peritumoral rCBV values of the glial tumors were significantly higher than those of metastatic ones, in accordance with the literature (*p* < 0.001 and *p* = 0.001, respectively).

Although the mean age of the patients with primary tumors was higher than that of patients with metastatic tumors (*p* = 0.001), the literature showed that mean age was not a significant factor affecting study heterogeneity for the perfusion MRI studies [5].

It has been shown that intratumoral susceptibility signals could help differentiate metastases from glial tumors due to the fact that glial tumors contain more intratumoral susceptibility signal foci than metastases in SWI sequences [24,25]. In accordance with the literature, the number of intratumoral susceptibility signal foci in SWI was significantly higher in the glial tumor group in our study.

Though there are studies in the literature investigating the role of DSC-MRI for the differentiation of brain metastases from glial tumors, to the best of knowledge, there is no past study comparing perfusion MRI measurements according to primary tumor origin. Our study contributes to the literature by further breaking down the metastatic brain masses based on the primary site of the tumor. Our study showed that metastatic brain masses originating from miscellaneous locations, including non-small-cell lung cancer, the gastrointestinal tract, colon cancer, ovarian tumors, malignant mesothelioma, and malignant epithelial salivary gland tumors, had statistically significant lower rCBV values compared to glial tumors (*p* < 0.05), and, although not statistically significant, there is a trend toward higher rCBV values in glial tumors compared to rCBV values of breast cancer, with median (IQR) tumoral rCBV values of 1.576 (0.914–2.912) for breast and 2.185 (1.25–2.8) for glial tumors (*p* = 0.009). Similarly, peritumoral rCBV values were significantly lower in metastatic brain tumors except for breast cancer metastasis compared to glial tumors. When the comparison was made by the type of the lung cancer metastasis, there was a statistically significant difference between high-grade glial tumors and non-small-cell lung cancer both in terms of peritumoral and tumoral rCBV (*p* = 0.003 and *p* < 0.001, respectively), while no significant difference was observed between high-grade glial tumors and small-cell lung cancer (*p* = 0.556 and *p* = 0.068, respectively). Based on this result, we suggest the use of DCS-MRI for the differentiation of brain tumor types, although it must be used with caution in patients with known breast cancer and small-cell-type lung cancer while deciding the nature of the tumor.

The current study also evaluated the diagnostic performance of the rCBV values for discriminating high-grade glial tumors from brain metastasis. For the specific cut-off rCBV values of 1.339 and 1.238 for tumoral and peritumoral areas, respectively, 87.5% and 58.3% sensitivity was recorded, with AUC values of 0.816 and 0.725, which means that rCBV measurements are highly effective in differentiating high-grade glial tumors from brain metastasis. Although variable cut-off values were reported in the literature for rCBV in patients with glial tumors and brain metastases [5], our cut-off values were similar to the cut-off values in the literature that used the same vendor as us. Past studies reported that perfusion MRI and rCBV measurement are relatively subjective due to the variabilities in parameters as well as the use of different vendors to scan the patients, and there is no consensus upon the optimum measurement technique [5]. Thus, cut-off values may vary according to the vendor that is used to acquire perfusion MRI.

Based on our study results, tumoral and peritumoral rCBV values of brain masses above specific cut-off points support the identification of glial tumors. We propose that this knowledge may help in determining the accurate radiological diagnosis of brain metastases from glial tumors, although this measurement tool must be used with caution in patients with breast cancer and small-cell-type lung cancer. Also, radiologists must be aware of the alterations in cut-off values based on the vendor used to scan the patients and adjust their approaches accordingly.

Multivariate analysis revealed that the occurrence of high-grade glial tumors increased with the increase in rCBV tumoral (OR: 2.321; 95% CI: 1.155–4.663; *p* = 0.018), and peritumoral rCBV values (OR: 5.07; 95% CI: 1.161–22.137; *p* = 0.031) were found to be independent risk factors. Based on this result, we propose the use of rCBV values for diagnosis as well as surgical or radiotherapeutic approaches to brain tumors as a complementary tool to conventional MRI studies.

The retrospective nature of the research and the small sample size were the limitations of our study. Also, while ROI sampling, tumoral necrosis and heterogeneity can be a barrier, especially when there is partial volume averaging. It is important to mention that the use of different vendors may influence the measurement values, and the lack of standardization of DSC perfusion techniques considering the different manufacturers and post-processing parameters are major restrictions for clinical implementations. A meta-analysis found that shorter TEs and smaller slice gaps were linked to higher pooled sensitivities [26]. Therefore, DSC perfusion MRI must be used as a complimentary tool in patients’ clinical settings and should not be thought of as a replacement for histological diagnosis.

## 5. Conclusions

DSC-MRI may be beneficial as a complementary technique to differentiate glial tumors and metastasis. Higher rCBV values stand out as a determinant of high-grade glial tumors. Compared to brain metastasis, breast cancer and small-cell-type lung cancer metastasis did not show statistically significant different rCBV values from high-grade gliomas; therefore, DSC-MRI must be used with caution in patients with known breast cancer and small-cell-type lung cancer when determining the nature of the tumor.

## Figures and Tables

**Table 1:** Patient characteristics.

Variables	All Patients (*n* = 95)
Age (years), *median* (*IQR*)	60 (51–67)
Gender, *n* (%)	
Female	42 (44.2)
Male	53 (55.8)
Tumor size, *median* (*IQR*)	20 (12–34)
Localization	
Global	29 (30.5)
Supratentorial	53 (55.8)
Infratentorial	13 (13.7)
Contrast enhancement, *n* (%)	
Diffuse	11 (11.6)
Heterogeneous	31 (32.6)
Punctate	9 (9.5)
Peripheral	44 (46.3)
Restricted diffusion, *n* (%)	38 (40)
SWI, microhemorrhage, *n* (%)	38 (40)
rCBV tumoral, *median* (*IQR*)	1.005 (0.663–2.032)
rCBV peritumoral, *median* (*IQR*)	0.915 (0.654–1.202)
Tumor, *n* (%)	
*Glial*	30 (31.6)
*Lung met*	40 (42.1)
*Breast met*	12 (12.6)
*Other met*	13 (13.7)
Tumor histopathology, *n* (%)	
*Low-grade glial tumor*	6 (6.3)
*High-grade glial tumor*	24 (25.3)
*Small-cell lung CA*	5 (5.3)
*Non-small-cell lung CA*	35 (36.8)
*Breast CA*	12 (12.6)
* *Miscellaneous*	13 (13.7)

* Miscellaneous includes colon carcinoma, gastrointestinal tract, carcinoma, ovarian tumor, malignant mesothelioma, and malignant epithelial salivary gland tumor; rCBV = relative cerebral blood volume; IQR = interquartile range; CA = carcinoma; met = metastasis.

**Table 2:** Characteristics of patients with glial tumors and brain metastases.

Variables	Brain Metastases (*n* = 65)	Glial Tumors (*n* = 30)	*p*
Age (years), *median* (*IQR*)	62 (53–69)	56 (45–60)	0.001
Gender, *n* (%)			
Female	25 (38.5)	17 (56.7)	0.097
Male	40 (61.5)	13 (43.3)	
Number of tumors, *n* (%)			
1 tumor	22 (33.8)	28 (93.3)	<0.001
2 tumors	9 (13.8)	2 (6.7)	
3 tumors	10 (15.4)	0 (0)	
4 tumors	7 (10.8)	0 (0)	
Multiple	17 (26.2)	0 (0)	
Tumor size, *median* (*IQR*)	15 (9–25)	35.5 (24–53)	<0.001
Localization			
Global	29 (44.6)	0 (0)	<0.001
Supratentorial	25 (38.5)	28 (93.3)	
Infratentorial	11 (16.9)	2 (6.7)	
Contrast enhancement, *n* (%)			
Diffuse	9 (13.8)	2 (6.7)	<0.001
Heterogeneous	11 (16.9)	20 (66.7)	
punctate	4 (6.2)	5 (16.6)	
Peripheral	41 (63.1)	3 (10)	
Restricted diffusion, *n* (%)	23 (35.4)	15 (50)	0.176
SWI, Microhemorrhage, *n* (%)	21 (32.3)	17 (56.7)	0.024
Tumoral rCBV, *median* (*IQR*)	0.879 (0.545–1.371)	2.185 (1.25–2.8)	<0.001
Peritumoral rCBV, *median* (*IQR*)	0.880 (0.651–1.017)	1.193 (0.849–1.714)	0.001

Mann–Whitney U test, Pearson chi-square test, Fisher’s Exact test; rCBV = relative cerebral blood volume; IQR = interquartile range; SWI = susceptibility-weighted imaging; *p* < 0.05 was considered as statistically significant.

**Table 3:** Tumoral and peritumoral relative cerebral blood volume of patients according to primary tumor location.

Variables, Median	Glial	Lung	Breast	Miscellaneous				
(IQR)	(*n* = 30)	(*n* = 40)	(*n* = 12)	(*n* = 13)	*p*	*p* ^1^	*p* ^2^	*p* ^3^
Tumoral rCBV	2.185 (1.25–2.8)	0.848 (0.467–1.04)	1.576 (0.914–2.912)	0.849 (0.508–1.339)	<0.001	<0.001	0.999	0.009
Peritumoral rCBV	1.193 (0.849–1.714)	0.886 (0.653–1.077)	0.944 (0.712–1.072)	0.692 (0.556–0.887)	0.004	0.041	0.999	0.004

Kruskal–Wallis test with post hoc Bonferroni correction. ^1^ Glial vs. lung, ^2^ glial vs. breast, ^3^ glial vs. other; *p* < 0.05 was considered as statistically significant; rCBV = relative cerebral blood volume; IQR = interquartile range.

**Table 4:** Discriminative performance of tumoral and peritumoral relative cerebral blood volume values in differentiating high-grade glial tumors from brain metastases.

			Cut-Off	Sensitivity	Specificity
Variables	AUC (95% CI)	*p*	Value	(%)	(%)
High-Grade Glial Tumor					
Tumoral rCBV	0.816 (0.720–0.890)	<0.001	>1.339	87.5	73.85
Peritumoral rCBV	0.725 (0.620–0.814)	0.003	>1.238	58.33	90.77

AUC: area under the curve, CI: confidence interval.; rCBV = relative cerebral blood volume; *p* < 0.05 was considered as statistically significant.

**Table 5:** Multivariate logistic regression analysis to determine factors associated with high-grade glial tumor.

Variables	Tumoral OR (95% CI)	*p*	Peritumoral OR (95% CI)	*p*
Age	0.91 (0.849–0.975)	0.008	0.924 (0.864–0.987)	0.019
Tumor size	1.049 (1.003–1.097)	0.037	1.04 (0.991–1.091)	0.109
Heterogeneous contrast enhancement	4.411 (0.947–20.545)	0.059	5.32 (1.121–25.255)	0.035
SWI, microhemorrhage	2.638 (0.563–12.359)	0.218	2.895 (0.63–13.296)	0.172
Tumoral rCBV	2.321 (1.155–4.663)	0.018	-	-
Peritumoral rCBV	-	-	5.07 (1.161–22.137)	0.031

OR = odds ratio; rCBV = relative cerebral blood volume; *p* < 0.05 was considered as statistically significant.

## Data Availability

The datasets analyzed during the current study are available from the corresponding author upon reasonable request and with permission from the University of Health Sciences Antalya Training and Research Hospital.
